# The effect of red blood cell transfusion on tissue oxygenation and microcirculation in severe septic patients

**DOI:** 10.1186/2110-5820-1-46

**Published:** 2011-11-08

**Authors:** Farid Sadaka, Ravi Aggu-Sher, Katie Krause, Jacklyn O'Brien, Eric S Armbrecht, Robert W Taylor

**Affiliations:** 1 St. John's Mercy Medical Center, St. Louis University, St. Louis, MO, USA

## Abstract

**Background:**

Microcirculation plays a vital role in the development of multiple organ failure in severe sepsis. The effects of red blood cell (RBC) transfusions on these tissue oxygenation and microcirculation variables in early severe sepsis are not well defined.

**Methods:**

This is a prospective, observational study of patients with severe sepsis requiring RBC transfusions of one to two units of non-leukoreduced RBCs for a hemoglobin < 7.0, or for a hemoglobin between 7.0 and 9.0 with lactic acidosis or central venous oxygen saturation < 70%. This study took place in a 54-bed, medical-surgical intensive care unit of a university-affiliated hospital. Thenar tissue oxygen saturation was measured by using a tissue spectrometer on 21 patients, and a vaso-occlusive test was performed before and 1 hour after transfusion. The sublingual microcirculation was assessed with a Sidestream Dark Field device concomitantly on 11 of them.

**Results:**

RBC transfusion resulted in increase in hemoglobin (7.23 (± 0.87) to 8.75 (± 1.06) g/dl; *p *< 0.001). RBC transfusion did not globally affect near-infrared spectrometry (NIRS)-derived variables. However, percent change in muscle oxygen consumption was negatively correlated with baseline (r = - 0.679, *p *= 0.001). There was no statistically significant correlation between percent change in vascular reactivity and baseline (*p *= 0.275). There was a positive correlation between percent change in oxygen consumption and percent change in vascular reactivity (r = 0.442, *p *= 0.045). In the 11 patients, RBC transfusion did not globally affect NIRS-derived variables or SDF-derived variables. There was no statistically significant correlation between percent change in small vessel perfusion and baseline perfusion (r = -0.474, *p *= 0.141), between percent change in small vessel flow and baseline flow (r = -0.418, *p *= 0.201), or between percent change in small vessel perfusion and percent change in small vessel flow (r = 0.435, *p *= 0.182).

**Conclusions:**

In a small sample population, muscle tissue oxygen consumption, microvascular reactivity and sublingual microcirculation were globally unaltered by RBC transfusion in severe septic patients. However, muscle oxygen consumption improved in patients with low baseline and deteriorated in patients with preserved baseline. Future research with larger samples is needed to further examine the association between RBC transfusion and outcomes of patients resuscitated early in severe sepsis, with an emphasis on elucidating the potential contribution of microvascular factors.

## Introduction

In the United States, approximately 750,000 cases of sepsis occur each year, of which at least 225,000 are fatal. One study that evaluated the epidemiology of sepsis between 1979 and 2000 demonstrated an 8.7% increase in the annual incidence of sepsis. The cost of management of one septic patient has been estimated at $50,000, amounting to annual costs of approximately $17 billion. Sepsis is the second-leading cause of death in noncoronary intensive care units (ICUs) and the tenth leading cause of death overall. Organ failure occurs in approximately one third of patients with sepsis and severe sepsis is associated with an estimated mortality rate of 30-50%. Seventy percent of patients with three or more organ failures (classified as severe sepsis or septic shock) die [1-8].

Red blood cell transfusion is one of the most commonly used interventions in the ICU to treat severe anemia, which often occurs in sepsis. In the United States, more than 14 million units of packed red blood cells (RBCs) are administered annually, many of which are administered in the ICU [9]. Approximately 40-80% of RBC transfusions in the ICU are not given for bleeding, but rather for low hemoglobin levels, for a decrease in physiological reserve, or for alterations in tissue perfusion [10,11]. In addition, RBC transfusion is recommended as part of early goal-directed therapy for patients with severe sepsis [12].

Patients with sepsis develop alterations in microvascular circulation, tissue oxygenation, and oxygen metabolism, all of which play a major role in the development of organ failure. Orthogonal polarization spectral (OPS) and sidestream dark field (SDF) imaging devices both provide high-contrast images of underlying microvasculature [13]. Using these devices, investigators have reported that the microcirculation is markedly altered in sepsis, alterations are more severe in nonsurvivors, and persistent microvascular alterations are associated with development of multiple organ failure and death [14-17]. The sublingual microcirculation has been the most extensively studied in patients with critical illness and sepsis.

Another noninvasive technique used is near-infrared spectrometry (NIRS) [18,19], which measures skeletal muscle tissue hemoglobin concentration and oxygen saturation before and after stagnant ischemia. Tissue ischemia is normally followed by arteriolar dilation and a temporary rise in local blood flow, a phenomenon termed reactive hyperemia (RH). RH is impaired in patients with severe sepsis [20,21]. Using NIRS, investigators have shown that oxygen consumption (during stagnant ischemia) and microvascular reactivity (RH) are altered in sepsis, are more severe in nonsurvivors, and persistence is associated with development of multiple organ failure and death [22-25].

The primary objective of this study was to evaluate the effect of RBC transfusion in severe septic patients on sublingual microvascular perfusion and flow using SDF and on muscle tissue oxygenation, oxygen consumption, and microvascular reactivity using NIRS. A secondary objective was to correlate the variables obtained from NIRS with those obtained from SDF.

## Methods

### Subjects

This prospective, observational study included 21 severe septic patients according to standard definition [26]. All patients received RBC transfusion for a hemoglobin < 7.0, or for a hemoglobin between 7.0 and 9.0 with lactic acidosis, or central venous oxygen saturation < 70%. All patients were clinically euvolemic (by CVP and/or echocardiogram) and in the first 12 hours of sepsis. Exclusion criteria included RBC transfusion in the preceding 72 hours, peripheral vascular disease, liver cirrhosis, age < 18 years, active bleeding, shock secondary to any other cause (cardiogenic, hemorrhagic, obstructive), and pregnancy. Hemodynamic, NIRS-derived, and SDF-derived variables were obtained immediately before (baseline) and 1 hour after transfusion of 1 unit of packed RBCs. During the study period, no bedside procedures were performed, doses of vasopressor and sedative agents were kept constant, and the patient's position in bed (head of bed at 30 degrees elevation) was not changed. This study was approved by the Institutional Review Board at St. John's Mercy Medical Center with waiver of written informed consent (# 09-953).

### Red blood cell transfusion characteristics

Packed red blood cell units were obtained from the blood bank (St. John's Mercy Medical Center). None of the RBC units transfused in this study were leukoreduced. Storage solution (saline-adenine-glucose-mannitol) was added to RBCs before storage. The storage period of RBCs is allowed up to 42 days.

### Measurements

The temperature, heart rate, arterial pressure, central venous pressure (when available), hemoglobin, central venous oxygen saturation, lactic acid, and arterial blood gases were recorded before and 1 hour after transfusion. The Acute Physiology and Chronic Health Evaluation II (APACHE II) score [27] was obtained at admission to the ICU, and the Sequential Organ Failure Assessment score [28] was obtained on the study day. The length of RBC storage before transfusion was noted in each case. NIRS measurements were obtained on all patients. SDF measurements were obtained for 11 patients. SDF measurements could not be obtained for all patients due to technical difficulties or safety concerns (i.e., some patients were not intubated, some were not sedated sufficiently).

### NIRS measurements and analysis

The thenar tissue oxygen saturation (StO_2_) and the tissue hemoglobin index (THI), an indicator of the blood volume in the region of the microvasculature sensed by the NIRS probe [29], were measured using a tissue spectrometer (InSpectra™ Model 650; Hutchinson Technology Inc., Hutchinson, MN, USA). This device uses reflectance mode probes to measure scattered light reflected at some distance from where the light is transmitted into the thenar muscle. Sample measurement signals were updated every 2 seconds.

During a period of hemodynamic stability (mean arterial pressure > 65 mmHg and no change in vasopressor doses for 2 hours), the NIRS probe was placed on the skin of the thenar eminence and a sphygmomanometer cuff was placed around the arm over the brachial artery. A large bore tube/cuff that inflates and deflates in less than one second was used to avoid cuff inflation and deflation from affecting the slope measurements. After a 3-minute period necessary to stabilize the StO_2 _signal, arterial inflow was stopped by inflation of the cuff to 50 mmHg above the systolic arterial pressure. After 3 minutes of ischemia the cuff pressure was released, and StO_2 _was continuously recorded for another 3 minutes (reperfusion period). Continuous measurements of the _StO2 _and THI were obtained during the vaso-occlusive test. Baseline StO_2 _and THI were recorded before the ischemic period and THI was recorded after 1 minute of occlusion. During occlusion, we calculated the StO_2 _desaturation slope (%/minute) obtained from the regression line of the first minute of StO_2 _decay after occlusion [29]. This is a representation of oxygen consumption. During the reperfusion phase, the StO_2 _upslope (%/second) was obtained from the regression line of the first 14 seconds of increased StO_2 _(seven StO_2 _values) following the ischemic period. This StO_2 _upslope of the reperfusion phase was used to quantify the intensity of the reactive hyperemic response following release of the occluding cuff. The percent change in recovery (upslope) was calculated as the difference between the StO_2 _upslopes of the reperfusion phase after and before transfusion divided by the StO_2 _upslope before transfusion. Muscle oxygen consumption (NIRVO2) was calculated as the product of the inverse value of the StO_2 _desaturation slope and the mean THI over the first minute of arterial occlusion [29] and is expressed in arbitrary units:

NIRVO2=(StO2desaturationslope-1)×(THIstartcuff+THI1min)]∕2

Percent change in NIRVO2 (downslope) was calculated as the difference between the NIRVO2 values after and before transfusion divided by NIRVO2 values before transfusion.

### SDF measurements and analysis

Sidestream dark field imaging was performed by using a handheld device that illuminates an area of interest. Light is emitted by a circle of light-emitting diodes. The reflected light is returned through the inner image-conducting core, which is optically isolated from the light-emitting diodes and caught on camera. Although assessing the microcirculation is based on light absorption by the hemoglobin contained in RBCs, this technique remains valid in anemia, as well as during acute changes in hemoglobin concentration [30]. Sidestream dark field imaging and semiquantitative analysis were performed as described in detail elsewhere [31]. In short, video images (Microscan; Microvision Medical, Amsterdam, the Netherlands) were captured via connection to a laptop computer. After the removal of saliva and other secretions using gauze, the device was gently applied (without significant pressure) to the lateral side of the tongue, in an area approximately 1.5-4 cm from the tip of the tongue. Three video recordings of 20 seconds in duration each at two time points (i.e., baseline and 1 hour posttransfusion) were analyzed by dividing the image into four equal quadrants. Quantification of flow (microvascular flow index-MFI) was scored per quadrant, for each size group of microvessel diameter: small (10-25 microns), medium (25-50 microns), and large (50-100 microns). Quantification of flow (0 = no flow, 1 = intermittent flow, 2 = sluggish flow, and 3 = continuous flow) was recorded. Microvascular flow index was calculated as the sum of each quadrant score divided by the number of quadrants in which the vessel type was visible. The final MFI was averaged over a maximum of 12 quadrants (three regions, four quadrants per region) derived from the overall flow impressions of all vessels with a particular range of diameter in a given quadrant. The heterogeneity index was calculated, following the method of Trzeciak and colleagues [16], as the difference between the highest and lowest MFI, divided by the mean MFI of all sublingual sites at a single time point. Calculation of total (small) vessel density was performed with the AVA 3.0 software package (MicroVision Medical, Amsterdam, The Netherlands), as described and validated recently [32] using a cutoff diameter for small vessels < 20 microns. After stabilization of the images using the AVA 3.0 software, we defined the perfused (small) vessel density (PVD) and the proportion of perfused (small) vessels (PPVs) in terms of the number and percentage of crossings with perfused (small) vessels per total length of three equidistant horizontal and three equidistant vertical lines (De Backer score), or as total length of perfused vessels divided by total surface of area (mm/mm^2^). To reduce observer measurement bias, sidestream darkfield images were analyzed off-line and in a blinded fashion by one of the investigators (FS), who was blinded to the patient's clinical course and the order of the sequences.

Percent change in PPV was calculated as the difference between the PPV values after and before transfusion divided by PPV values before transfusion. Percent change in MFI was calculated as the difference between the MFI values after and before transfusion divided by MFI values before transfusion.

### Analysis

Descriptive statistics were performed for the full and subgroup samples to assess similarities in patient characteristics, including age, gender, source of infection(s), age of blood, APACHE II score, and discharge status (i.e., mortality). Changes in hemodynamic and other observed measurements taken before (pre) and 1 hour after (post) the transfusion were assessed by a paired *t *test. Mean, standard deviation and *p *value were reported for each comparison. Analysis for the full sample and subgroup were conducted separately. A Pearson correlation coefficient (PCC) was calculated to describe the association between percent change in NIRVO2 (downslope), baseline NIRVO2 (downslope), percent change in recovery (upslope), and baseline upslope using subjects in the full sample. This method was repeated for the subgroup with additional comparisons, including percent change in PPV for small vessels vs. baseline PPV, percent change in MFI, percent change in NIRVO2 (downslope), and percent change in recovery (upslope). Percent change in MFI for small vessels was correlated with baseline MFI, percent change in NIRVO2 (downslope) and percent change in recovery (upslope). All analyses were conducted with SPSS/PASW version 18 (Chicago, IL) by an investigator (EA) who was not involved with data collection or analysis of sidestream darkfield images.

## Results

The study included 21 severe septic patients with NIRS-derived data (full sample), 11 of whom also had SDF-derived data (subgroup sample; Table 1). The median APACHE II scores were 24 and 25 for the full sample and the subgroup sample respectively, and in-hospital mortality was 47.6% and 45.6%, respectively. No transfusion-related adverse reactions were observed during the study. The mean arterial pressure increased from 69.67 mmHg (± 8.76 mmHG) to 73.52 mmHg (± 11.08 mmHg; *p *= 0.08) in the full sample, and from 67.36 mmHg (± 7.97 mmHG) to 73.18 mmHg (± 12.16 mmHg; *p *= 0.02) in the subgroup sample (Table 2). The median RBC storage time was 32 days (21-39) in the full sample and 32 days (22-39) in the subgroup sample.

**Table 1:** Characteristics of the study groups

	Full sample^a^ (n = 21)	Subgroup^b^ (n = 11)
Age (yr)	71 (41-87)	73 (55-83)
Male gender, %	11 (52.4)	5 (45.5)
APACHE II score	24 (17-39)	25 (20-39)
SOFA score	8 (3-17)	9 (3-16)
Source of infection, %		
Lung	11 (52.4)	3 (27.3)
Abdomen	6 (28.6)	4 (36.4)
Urinary tract	3 (14.3)	3 (27.3)
Line	1 (4.7)	1 (9.0)
Vasopressors/inotropes dose^c^		
Norepinephrine, mcg/min	10; 10 (2-40)	6; 10 (2-25)
Dobutamine, mcg/kg/min	4; 5 (2.5-10)	2; 3.7 (2.5-5)
Sedation/analgesic dose^c^		
Midazolam, mg/hr	7; 2 (2-4)	4; 2 (2-4)
Fentanyl, mcg/hr	8; 100 (50-400)	4; 100(50-400)
Human recombinant activated protein C, %	14.3	27.3
Renal replacement therapy, %	33.3	27.3
Red blood cell storage time (days)	32 (21-39)	32 (22-39)
In-hospital mortality, %	47.6	45.6

Data are presented as median (25th to 75th percentiles) or *n *(%).

APACHE, Acute Physiology and Chronic Health Evaluation; SOFA, Sequential Organ Failure Assessment.

^a^Full sample, all with NIRS data.

^b^Subgroup, all have both NIRS and SDF data.

^c^n; dose.

**Table 2:** Physiologic and near-infrared spectroscopy-derived variables before and 1 hour after red blood cell transfusion

	Full Sample				Subgroup			
		Baseline	After Transfusion			Baseline	After Transfusion	
	n	Mean (SD)	Mean (SD)	*p*	n	Mean (SD)	Mean (SD)	*p*
Hemoglobin (g/dl)	21	7.2 (0.8)	8.7 (1.1)	0.00	11	7.5 (0.8)	8.9 (1.1)	0.00
Heart Rate (beats/min)	21	91 (15)	91 (15)	0.34	11	91 (18)	89 (18)	0.14
Temperature (°F)	21	97.8 (1.2)	97.7 (1.2)	0.65	11	98.1 (1.2)	98.1 (1.2)	0.96
Mean arterial pressure (mmHg)	21	69.6 (8.7)	73.5 (11.1)	0.08	11	67.3 (7.9)	73.2 (12.1)	0.02
Central venous pressure (mmHg)	12	16 (5.7)	16.2 (4.3)	0.79	7	15.2 (4.3)	16.1 (5)	0.34
Lactate (mmol/l)	12	4.1 (3.5)	3.9 (3.4)	0.47	6	3.7 (2.1)	3.8 (2.4)	0.73
Arterial partial pressure of oxygen (mmHg)	6	124.6 (97.6)	95.2 (29.2)	0.49	3	164 (137.1)	101 (38)	0.5
pH	6	7.3(0.1)	7.3 (0.1)	0.62	3	7.3(0.1)	7.3(0.1)	0.24
Central venous oxygen saturation (%)	10	59.1 (9.2)	63.8 (8.8)	0.11	6	62.3 (9.2)	64.6 (8.9)	0.48
SaO_2_/FiO_2_	21	264.1 (114.8)	270.9 (97.2)	0.46	11	249.5 (105.9)	259.1 (91.5)	0.19
Thenar tissue oxygen saturation (%)	21	76.2 (9.3)	75.8 (8.1)	0.80	11	76.8 (8.4)	75.8 (8.8)	0.69
Tissue hemoglobin index (arbitrary units)	21	10.7 (3.4)	12.2 (3.5)	0.01	11	10.9 (3.1)	12.2 (4.1)	0.07
Thenar tissue oxygen saturation upslope of the reperfusion phase (%/second)	21	2.5 (1.3)	2.6 (1.5)	0.39	11	2.2 (1)	2.1 (1.1)	0.78
Muscle oxygen consumption (arbitrary units)	21	113.6 (56.43)	124.1 (43.6)	0.26	11	104.4 (41.1)	112.5 (40.3)	0.5

### Full sample

**Figure 1:**
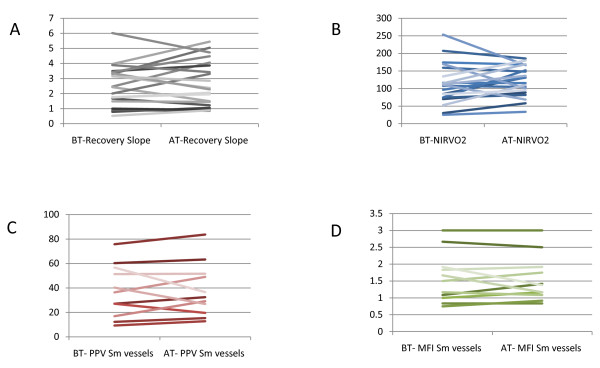
**Tissue oxygenation and microcirculation variables for individual patients from before and after transfusion. A** Recovery slopes for individual patients from before and after transfusion for full sample. ** B **NIRVO2 for individual patients from before and after transfusion for full sample. **C **PPV small vessels for individual patients from before and after transfusion for subgroup sample. **D **MFI small vessels for individual patients from before and after transfusion for subgroup sample.

**Figure 2:**
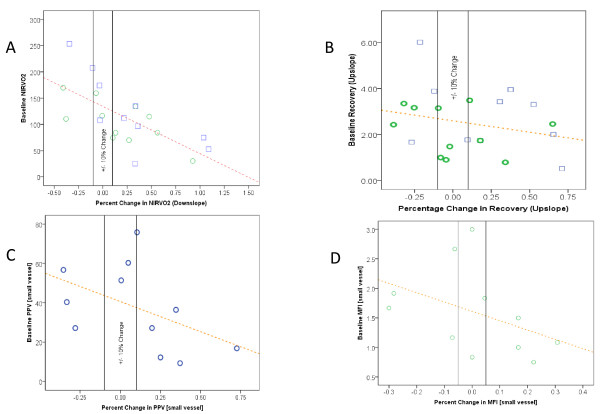
**Tissue oxygenation and microcirculation variables: relationship between baseline and percent change from before and after transfusion. A** Tissue oxygen consumption (NIRVO2) significantly correlates positively with microvascular reactivity (recovery - upslope) in both the full sample (r = 0.442, p = 0.045) and in the subgroup sample (r = 0.775, p = 0.005). ** B **Relationship between baseline recovery (upslope) and percent change in recovery (upslope) for both the full sample (r = -0.25, *p *= 0.275) and subgroup sample (r = -0.278, *p *= 0.407). **C **Relationship between baseline small vessel perfusion (PPVsmall vessel) and percent change in small vessel perfusion for the subgroup sample (r = -0.474, *p *= 0.141). **D **Relationship between baseline small vessel flow (MFI small vessel) and percent change in small vessel flow in the subgroup sample (r = -0.418, *p *= 0.201).

**Figure 3:**
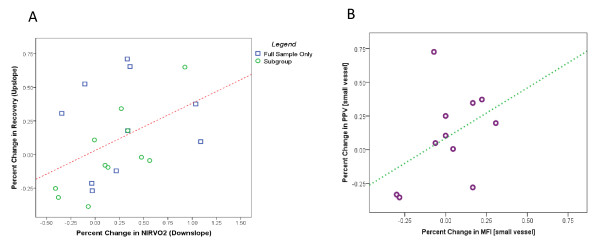
**Correlations among Tissue oxygenation variables and Microcirculation variables. A** Tissue oxygen consumption (NIRVO2) significantly correlates positively with microvascular reactivity (recovery - upslope) in both the full sample (r = 0.442, p = 0.045) and in the subgroup sample (r = 0.775, p = 0.005).  **B **Small vessel Microvascular Flow Index (MFIsmall--marker of flow) correlates positively with proportion of perfused small vessels (PPVsmall--marker of perfusion) in the subgroup sample (not statistically significant; r = 0.435, *p *= 0.182).

In the full sample, blood transfusion resulted in increase in hemoglobin (7.23 g/dl (± 0.87 g/dl) to 8.75 g/dl (± 1.06 g/dl; *p *< 0.001; Table 2). Red blood cell transfusion did not globally affect NIRS-derived variables (Table 2; Figure 1A,B). However, percent change in NIRVO2 was negatively correlated with baseline NIRVO2 (r = -0.679, *p *= 0.001; Figure 2A). There was no statistically significant correlation between percent change in recovery (upslope) and baseline recovery upslope (*p *= 0.275; Figure 2B). There was a positive correlation between percent change in NIRVO2 and percent change in the recovery upslope (r = 0.442, *p *= 0.045; Figure 3A).

### Subgroup sample

In the subgroup sample, blood transfusion resulted in increase in hemoglobin (7.48 g/dl (± 0.83 g/dl) to 8.95 g/dl (± 1.12 g/dl); *p *< 0.001; Table 2). Red blood cell transfusion did not globally affect NIRS-derived variables or SDF-derived variables (Tables 2 and 3; Figure 1C,D). Similar to the full sample, percent change in NIRVO2 was negatively correlated with baseline NIRVO2 (r = -0.689, *p *= 0.019; Figure 2A). There was no statistically significant correlation between percent change in the recovery upslope and baseline recovery upslope (*p *= 0.407; Figure 2B). There was a positive correlation between percent change in NIRVO2 and percent change in the recovery upslope (r = 0.775, *p *= 0.005; Figure 3A). These findings suggest that the subgroup sample is similar in most observable regards to the full sample.

**Table 3:** Sidestream Dark Field-derived microcirculatory variables before and 1 hr after red blood cell transfusion

Subgroup	Baseline			After transfusion	
Measurement	Vessel size	n	Mean (SD)	Mean (SD)	*p*
Total vessel density (mm/mm^2^)	Small	11	22.4 (5.9)	21.5 (5.5)	0.36
Total vessel density (mm/mm^2^)	Large	11	3.4 (1.3)	3.9 (1)	0.2
Total vessel density (mm/mm^2^)	All	11	25.7 (6.4)	25.4 (6)	0.73
Perfused vessel density (mm/mm^2^)	Small	11	9.5 (4.8)	9.4 (4.8)	0.91
Perfused vessel density (mm/mm^2^)	Large	11	3 (1.5)	3.7 (1.2)	0.09
Perfused vessel density (mm/mm^2^)	All	11	12.5 (5.4)	13.3 (4.7)	0.53
Proportion of perfused vessels (%)	Small	11	37.6 (21.5)	38.2 (21.8)	0.85
Proportion of perfused vessels (%)	Large	11	100	100	1
Proportion of perfused vessels (%)	All	11	51.6 (23.8)	53.9 (20.9)	0.45
De Backer score (n/mm)		11	14.7 (3.8)	14.8 (3.5)	0.91
Microvascular Flow Index	Small	11	1.6 (0.7)	1.6 (0.7)	0.76
Microvascular flow index	All	11	2.3 (0.4)	2.4 (0.3)	0.3
Heterogeneity index (%)		11	0.3 (0.2)	0.4 (0.3)	0.19

### THI results

THI variables behaved exactly similar to StO2 variables (data not shown). THI correlated with StO_2_. For example, in the full sample before transfusion, THI positively correlated with StO_2 _(r = 0.47, *p *= 0.03). THI increased after transfusion in the full sample (Table 2), but not in the subgroup sample. There was no correlation between THI and hemoglobin levels before transfusion (r = 0.11, *p *= 0.64) or after transfusion (r = 0.16, *p *= 0.49).

### Correlations between variables from NIRS and SDF

There was no statistically significant correlation between percent change in small vessel PPV and baseline small vessel PPV (r = -0.474, *p *= 0.141; Figure 2C). There was no statistically significant correlation between percent change in small vessel MFI and baseline small vessel MFI (r = -0.418, *p *= 0.201; Figure 2D). There was no statistically significant correlation between percent change in small vessel PPV and percent change in small vessel MFI (r = 0.435, *p *= 0.182; Figure 3B). Although there was no significant correlation between NIRS-derived variables (NIRVO2, recovery upslope) and SDF-derived variables (PPV, MFI), all changes in NIRS-derived variables occurred in the same direction as SDF-derived variables (Figures 2 and 3).

## Discussion

The main finding of our study was that RBC transfusion had no global effect on muscle oxygen saturation, oxygen consumption, microvascular reactivity, vessel perfusion, or microvascular flow in severe septic patients. However, there was considerable variance between subjects. There was an improvement in oxygen consumption in patients with altered oxygen consumption at baseline and deterioration in oxygen consumption in patients with preserved baseline oxygen consumption.

Prospective studies in ICU patients showed a higher mortality rate in patients receiving RBCs than in those not receiving RBCs. These results suggest that a more restrictive transfusion strategy was safe in the ICU population and might be beneficial for some patients [33,34]. Guidelines published as part of the Surviving Sepsis Campaign [12] have endorsed use of RBCs in the treatment of patients with severe sepsis who show evidence of hypoperfusion. This recommendation is primarily based on data published by Rivers et al. [35] who evaluated a bundle approach to patients in severe sepsis. Red blood cell transfusion to obtain a hematocrit of 30% is included in this bundle for patients with a central venous oxygen saturation < 70%. Patients achieving this goal had better outcomes than patients who did not reach the goal. The specific effect of transfusion was not evaluated in this study; however, because the investigation was designed to assess the overall bundle rather than its component parts. Using NIRS or SDF, several investigators have reported that microcirculation is markedly altered in sepsis, that these alterations are more severe in nonsurvivors than in survivors, that persistent microvascular alterations are associated with development of multiple organ failure and death, and that microvascular alterations are the most sensitive and specific predictor of outcome in septic patients [14-17,22-25]. Our goal was to study the effect of RBC transfusion on microvascular variables in severe septic patients using both NIRS and SDF.

The effects of RBC transfusion on the microcirculation in sepsis could be numerous. Several studies have demonstrated that RBC rheology is impaired (increased aggregation, decreased deformability, alterations of RBC shape) in sepsis [36-39]. These alterations could contribute to the microcirculatory alterations observed in critically ill patients [39]. RBC also can act as oxygen sensor, which can modulate tissue oxygen flow variables - by the release of the vasodilators, nitric oxide [40,41], or ATP [42]. This release of vasodilators from RBCs during hypoxia could be impaired during storage and/or sepsis. Storage of RBCs decreases levels of 2,3-diphosphoglycerate and adenosine triphosphate (ATP) levels with a resultant increase in oxygen affinity and a decrease in the ability of hemoglobin to offload oxygen. Morphological changes in erythrocytes occur during storage which may result in increased fragility, decreased viability, and decreased deformability of red blood cells. A release of a number of substances occurs during storage resulting in such adverse systemic responses as fever, cellular injury, alterations in regional and global blood flow, and organ dysfunction. Several studies have demonstrated that transfusion with RBCs that have been stored for long time periods is associated with poorer oxygen delivery than is transfusion with fresher cells [43-49]. The median RBC storage time in our study was 32 days, which is similar to other studies. A recent literature review reported no strong association between duration of storage and complications [50]. In addition, Creteur et al. [51] using NIRS and Sakr et al. [52] using OPS showed that RBC storage time had no influence on the microvascular response to red blood cell transfusion.

Our study differs from Creteur et al. in several points. We studied severe septic patients, whereas Creteur et al. studied hemodynamically stable patients, 41% of whom had sepsis. We transfused older (median RBC storage time = 32 vs. 18 days) RBCs; ours were all nonleukoreduced, whereas theirs were all leukoreduced. We used both NIRS and SDF in our study, whereas they only used NIRS. Our study also differed from Sakr et al. We transfused older blood (median RBC storage time = 32 vs. 24 days); ours were all nonleukoreduced, whereas theirs were all leukoreduced. We used both NIRS and SDF in our study, whereas they only used OPS (older version of SDF). In a very recent review on monitoring the microcirculation in critically ill patients, De Backer et al. concluded that a monitoring device should be able to detect capillary perfusion, flow, and heterogeneity of perfusion. This is best achieved with handheld microvideoscopic techniques, such as OPS and SDF. They also concluded that the use of vascular occlusion tests with laser Doppler or NIRS investigates microvascular reactivity, another important, but different, aspect of microvascular function. De Backer suggested that "Combining techniques may be of interest in the future" [53]. To our knowledge, our study is the only human study that employed both techniques in monitoring the impact of an intervention on the microcirculation. Each of these three studies showed similar findings. Creteur et al. demonstrated an improvement in microvascular reactivity and tissue oxygen consumption in patients with altered microvascular reactivity and tissue oxygen consumption at baseline and deterioration in microvascular reactivity and tissue oxygen consumption in patients with preserved baselines [51]. Sakr et al. showed an improvement in sublingual microvascular perfusion in patients with altered perfusion at baseline and deterioration in sublingual microvascular perfusion in patients with preserved baseline perfusion [52]. All showed no global effect of RBC transfusion on the microvascular variables.

In a recent study that evaluated perioperative RBC transfusions in patients who underwent cardiac surgery using SDF and sublingual reflectance spectrophotometry, Yuruk et al. showed that RBC transfusion improved sublingual microcirculatory density, but not perfusion velocity, and improved microcirculatory oxygen saturation [54]. Their study included a totally different patient population, patients with (relatively) healthy microcirculation.

Why do some patients show beneficial effects of RBC transfusions while others do not? Friedlander et al. observed that RBC transfusions improved RBC deformability in patients with sepsis, probably by replacing rigid, endogenous RBCs by less dysfunctional, exogenous RBCs [55]. Transfusions may therefore be deleterious when performed in patients with preserved deformability, vasoreactivity, perfusion, and/or flow but may be favorable when performed in patients in whom these variables are markedly altered.

Interestingly, RBC transfusion-induced changes in NIRVO2, in the recovery upslope of the reperfusion phase, in PPV, and in MFI were all in the same direction, suggesting that an improvement or worsening in microvascular reactivity, microvascular perfusion, and microvascular blood flow may be associated with an increase or decrease in local muscle oxygen consumption, respectively.

NIRS-derived variables showed changes in the same direction compared with SDF-derived variables (Figures 2 and 3). These changes were not, however, statistically significant. This is likely secondary to a small sample size. In fact that these two devices monitor different aspects of the microvasculature, as well as different organs also may have contributed. Hence, using both devices may be complimentary and a point of strength for this study.

Our study has its limitations. Our small sample size and the fact that some variables could not be obtained in some patients is an obvious limitation. The limited number of patients does not make it possible to determine whether initial deranged microcirculatory parameters could really influence the final response to RBC transfusion. NIRS monitors hemoglobin oxygen saturation in arterioles, venules, and capillaries in the measured volume of tissue, and the relative contributions of arterial, venous, and capillary blood within the measured volume of tissue cannot be determined. NIRS does not measure microcirculatory blood flow or perfusion. It also targets muscle tissue, specifically the thenar muscle. SDF monitors the capillaries and venules (not arterioles), but this device monitors the actual flow and perfusion and their heterogeneity in the microvessels. SDF data could be analyzed only semiquantitatively. SDF targets the sublingual mucosa, which shares a similar embryonic origin with the digestive mucosa (always involved pathologically in sepsis) but may not reflect other microcirculatory beds. Our measurements were restricted to 1 hr after RBC transfusion, therefore, later alterations due to transfusion may have been missed. However, longer follow-up periods are practically difficult because of inevitable changes in therapy and procedures in these critically ill patients that could themselves affect the microcirculation and other outcomes. THI increased after transfusion in the full sample (Table 2), which could alter the NIRVO2 measurements (refer to NIRS measurements and analysis above). THI does not reflect systemic hemoglobin levels as a result of Fahraeus effect, heterogeneous flow distributions, and local conditions (such as vasoconstriction and edema) [56,57]. In addition, Doerschug et al. showed that the THI was not related to blood hemoglobin concentration in patients with severe sepsis [22]. Similarly, in our study, there was no correlation between THI and hemoglobin levels before transfusion (r = 0.11, *p *= 0.64) or after transfusion (r = 0.16, *p *= 0.49). Moreover, despite the increase in THI in the full sample, there was an improvement in NIRVO2 in patients with altered baseline and deterioration in NIRVO2 in patients with preserved baseline in both the full sample and the subgroup sample, suggesting that this relationship is real. Because StO_2 _represents the average of the hemoglobin oxygen saturation in arterioles, venules and capillaries in the whole tissue sample, NIRS is not able to demonstrate changes on microvascular density or heterogeneity. As a result, we must continue to explore the meaning of reactive hyperemia as a surrogate of microvascular functionality.

## Conclusions

The effects of RBC transfusions on microvascular oxygenation, consumption, reactivity, perfusion, and flow are quite variable and may be dependent on baseline values. In this observational study of limited size, no effect of RBC transfusion on any measured microcirculation variables in severe septic patients was observed. This study does suggest that better means of identifying the need for transfusion are needed and that blindly transfusing to an arbitrarily set (and high) Hb may be detrimental. This study involves a small sample of patients, based on which strong recommendations cannot be made. Future research with larger samples is needed to further examine the association between RBC transfusion and outcomes of patients resuscitated early in severe sepsis, with an emphasis on elucidating the potential contribution of microvascular factors.

## Competing interests

The authors declare that they have no competing interests.

## Financial/nonfinancial disclosures

All authors report that no potential conflicts of interest exist with any companies/organizations whose products or services may be discussed in this article.

## Authors' contributions

FS contributed to conceiving the study, acquiring and managing the data, analyzing the data and interpreting the results, drafting and revising the manuscript, and approving the manuscript in its final form. RA, KK, and JO contributed to acquiring and managing the data, revising the manuscript, and approving the manuscript in its final form. EA contributed to performing statistical analysis, acquiring and managing the data, revising the manuscript, and approving the manuscript in its final form. RT contributed to analyzing the data and interpreting the results, revising the manuscript, and approving the manuscript in its final form.
